# Perceived Risk of Mental Health Problems in Primary Care

**DOI:** 10.3389/fnagi.2015.00212

**Published:** 2015-11-17

**Authors:** Constança Paúl, Laetitia Teixeira, Maria João Azevedo, Sara Alves, Mafalda Duarte, Rónán O’Caoimh, William Molloy

**Affiliations:** ^1^Research and Education Unit on Ageing, Institute of Biomedical Sciences Abel Salazar, University of PortoPorto, Portugal; ^2^Centre for Gerontology and Rehabilitation, St Finbarrs Hospital – University College CorkCork City, Ireland; ^3^COLLaboration on AGEing, University College Cork, Cork, and NetwellCASALA – Dundalk Institute of TechnologyDundalk, Ireland; ^4^Health Research Board, Clinical Research Facility Galway, National University of IrelandGalway, Ireland

**Keywords:** risk, primary care, old people, mental health, RISC

## Abstract

In the face of limited resources and an aging population with increasingly care needs, healthcare systems must identify community-dwelling older adults with mental health problems at higher risk of adverse outcomes such as institutionalization, hospitalization and death, in order to deliver timely and efficient care. The objectives of this study were to assess the prevalence of mental health concerns and the associated perceived risk of adverse outcomes in a large sample of older patients in primary care (PC). We trained general practitioners and nurses to use the Risk Instrument for Screening in the Community to rank perceived risk of mental health concerns (including neurocognitive and mood disorders) from 1 (mild) to 3 (severe). The mean age of the 4499 people assessed was 76.3 years (*SD* = 7.3) and 2645 (58.8%) were female. According to the PC team 1616 (35.9%) were perceived to have mental health concerns of whom 847 (52.4%) were mild, 559 (34.6%) were moderate and 210 (13%) were severe. Patients with mental health concerns had higher odds of perceived risk of adverse outcomes (OR = 2.22, 95% CI 1.83–2.69 for institutionalization; OR = 1.66, 95% CI 1.41–1.94 for hospitalization; OR = 1.69, 95% CI 1.42–2.01 for death). These results suggest a high prevalence of mental health concerns among older adults and supports the need for early identification of patients at high-risk of adverse healthcare outcomes.

## Introduction

There is growing recognition of the need to develop inclusive health service policies to integrate and track mental health issues. Previously surrounded by misconceptions and stigma, the importance of good mental health is now widely accepted. Reasons to promote the integration of mental health issues into primary care (PC) include: “(1) The burden of mental disorders is great; (2) Mental and physical health problems are interwoven; (3) The treatment gap for mental disorders is enormous; (4) PC for mental health enhances access; (5) PC for mental health promotes respect of human rights; (6) PC for mental health is affordable and cost-effective; (7) PC for mental health generates good health outcomes" (WHO and WONCA, 2008). More recently, the World Health Assembly recognized the extent to which mental and neurological disorders including Alzheimer’s disease can cause morbidity and subsequent disability (WHA, 2012).

Mental health disorders including neurocognitive disorders affect approximately 165 million (38.2%) people in the European Union (EU), with no substantial cultural or country variations for most conditions (Wittchen et al., 2011). Mental health disorders contribute to 26.6% of the total burden of disease [disability adjusted life years (DALY)] in Europe (Wittchen et al., 2011). Recognizing the differences between study methods, the lifetime prevalence of mental health problems may still be as high as 57%, particularly in lower socioeconomic groups (Rabins et al., 1996). Excluding dementia, the prevalence of psychiatric disorders is estimated at 16.3%, potentially rising to 21.6% by the year 2030 (Jeste et al., 1998). Psychiatric disorders are underestimated because of cognitive impairment, physical disorders, ageism or because older people attribute feeling depressed to old age itself and are discouraged from seeking help or reporting symptoms (Sarkisian et al., 2003). Others have subclinical disorders that do not meet the diagnostic criteria for psychiatric disorders but require attention (Xavier et al., 2013), contributing significantly to morbidity and mortality (Gallo et al., 1997; Steffens et al., 2000). The prevalence of these disorders approaches 33% in people 70 years and over (the Berlin Aging Study) (Helmchen et al., 1999). In Portugal, the annual prevalence of mental disorders is 22.9%, with 7.3% reported as mild, 11.6% as moderate and 4% as severe (Caldas de Almeida and Xavier, 2013). The Portuguese National Inquiry on Health (INE, 2009) showed that there is a high prevalence of probable psychological distress in older people that varies from 40.6% in people aged 65–74 years to 42.2% in those aged 75–84 years, reducing to 36.3% by 85 years. The percentage of women with psychological suffering is higher than for man, affecting half of older Portuguese women. These data are in line with international comparative studies that show that Portugal ranks high compared to other nations (Caldas de Almeida and Xavier, 2013).

Mental and physical health problems are often interwoven, particularly in older people (WHO and WFMH, 2010). The bidirectional negative impact of physical disease and depression may result in further disability (Menchetti et al., 2001) and is independently associated with an increased risk of mortality (Ganguli et al., 2002).

In high-income countries between 35 and 50% of people receive insufficient treatment (WHA, 2012), often due to under-detection and/or inadequate access resulting in a large treatment gap for mental disorders. Utilizing PC resources is crucial to overcome this, particularly in Portugal where 17% of patients with mental health problems seek help in PC compared to 11% in other EU countries (EC, 2010). Training health professionals to recognize mental health concerns by providing them with efficient and effective instruments is a first step to insure that older people can get proper treatment for their condition (WHA, 2012). Four theoretical models of managing mental health disorders in PC include: training; consultation-liaison; collaborative care, and replacement/referral (Bower and Gilbody, 2005). The training of PC professionals to identify and manage risk factors seems most appropriate.

Another important component is to support patients with mental health disorders in their home environment by supporting their caregivers or social network. Caregivers (mainly family) are considered the backbone of long-term care systems (OECD, 2013). The positive effect of an informal carer is well-recognized in mental health, particularly where the caregiver assumes the role of confident. On the other hand, caregiver strain or burden increases the risk of adverse healthcare outcomes (Paúl and Martin, 2003; Carretero et al., 2009). The number of people aged 50 or more years reporting to be caregivers in 2010 was 15.6% with 62.3% being women and 87% providing care on a daily basis (OECD, 2013). In Portugal, as in most of the southern European countries, there is a strong culture of family providing care for their older relatives, mainly supported by women (Lopes, 2013).

Given the importance of integrating mental health services into PC by efficiently and effectively screening and triaging patients with mental health disorders, this paper aims to: (i) establish the point prevalence of mental health concerns among community-dwelling older adults in PC in northern Portugal, (ii) identify predictive factors associated with mental health concerns, and (iii) determine the extent that mental health concerns affect patients perceived risk of three adverse healthcare outcomes: institutionalization, hospitalization and death as perceived by healthcare professionals working in the community using a new, short, global subjective risk prediction instrument called the Risk Instrument for Screening in the Community (RISC; O’Caoimh et al., 2014, 2015c).

## Materials and Methods

### Study Design

This paper presents cross sectional data from a large, on going, prospective cohort study on mental health in northern Portugal. The project was approved by the ethics committee of the Regional Association of Health North (ARS North) and by each of the 24 Associations of Health Centers in the region where data was collected.

### Participants

The sample comprises 4499 consecutive patients attending 29 PC practices who met the following inclusion criteria: (i) aged > = 65 years-old; (ii) living in the community; (iii) PC patients; (iv) living in the area covered by the ARS North; (v) provided informed consent. The exclusion criteria included patients aged less than 65 years, those deemed to be actively dying, including those receiving palliative care, and those already in institutional care, i.e., nursing home residents. Those not attending the PC center regularly, i.e., those lost to regular follow-up/attendance were also excluded as no accurate demographic data were available and they could not be scored with the RISC.

### Measures

The RISC is a short (2–5 min), reliable (O’Caoimh et al., 2012, 2014) and valid global subjective assessment of risk. It has good internal consistency (O’Caoimh et al., 2015b,c). It is used as a pre-screen stratifying patients according to their risk level and is scored with a five point Likert scale from 1 to 5, where 1 is the lowest risk and 5 is the highest of three adverse health care outcomes (institutionalization, hospitalization, and death) at 1 year from assessment. Each outcome is scored separately. The RISC also collects demographic data and scores the ability of the caregiver network (both formal and informal) to manage risk across three domains: the patients’ Mental State, ADL State and Medical State, which can be used to inform the subjective assessment. The caregiver network score is again presented as a five point Likert scale, scored from 1 (can manage all risks) to 5 (the caregiver network is a liability or is absent). Risk is determined by a subjective assessment based upon the information gathered such that: risk equates to the severity of the concern minus the protective effect of the caregiver network for each of the three domains. The RISC is available at http://www.biomedcentral.com/1471-2318/14/104/figure/F1. Patients can be grouped into minimum (RISC scores 1 and 2) and maximum-risk (RISC scores 3–5) to facilitate analysis.

The RISC was developed in University College Cork (UCC), Ireland, as part the Community Assessment of Risk Treatment and Strategies (CARTS) program, a component of Irelands successful three star reference site application under the European Innovation Partnership on Active and Healthy Ageing (Sweeney et al., 2013; O’Caoimh et al., 2015d). The RISC was initially validated in Ireland against the Clinical Frailty Scale (CFS; Rockwood et al., 2005), an established frailty instrument, in 803 community dwelling older adults, aged over 65 years (O’Caoimh et al., 2014, 2015c; Leahy-Warren et al., 2015). Public health nurses scored the RISC and CFS for each patient and independent investigators, blind to the RISC score, followed up individual outcomes at 1 year. Those classified as maximum-risk were significantly more likely to experience all three outcomes (institutionalization, hospitalization, and death) at 1 year. The RISC had greater accuracy compared with the CFS, albeit it was a non-significant difference. The RISC better predicted institutionalization and death than hospitalization. A recent systematic review of risk-prediction instruments in the community confirmed that the risk compares favorably with similar tools, all of which have poor accuracy in predicting hospitalization (O’Caoimh et al., 2015a).

### Procedures

The RISC was firstly translated from English into Portuguese by a committee of three experts in gerontology, all fluent in English. The Portuguese version of the RISC was then back translated into English. To ensure semantic equivalence and acceptability, this process was performed by a professional English translator and by a professional with experience in gerontology. The draft version was discussed with the team of authors and some minor changes were made. The instrument was piloted by a group of five healthcare professionals to ensure comprehensibility and no further changes were introduced.

Healthcare professionals, general practitioners (GPs) and practice nurses who agreed to participate, received 4 h of training and certification in scoring the RISC, delivered by the Portuguese research team that had itself received a 2-day training session from the authors of the RISC in UCC, Ireland. RISC training introduces the concept of risk and adverse outcomes; it discusses the main areas of concern (mental, functional, and medical), its relevance for the assessment, its contents and scoring instructions. RISC training is shown to increase inter-rater reliability (O’Caoimh et al., 2012). Once trained, GPs and a small number of practice nurses scored the RISC on their own patients only, using their clinical knowledge of each patient’s current health status.

### Statistical Analysis

Descriptive statistics were used to characterize the sample. Potential predictive factors relating to mental health concerns were tested using univariable logistic regression models. Considering covariates statistically significant in univariable models, a multivariable logistic regression model was performed.

Additionally, the effect of the presence/absence of mental health concerns, the severity and the ability of the caregiver network were tested as potential predictive factors of the risk of each adverse outcome (institutionalization, hospitalization, and death) in three distinct multivariable logistic regression models (adjusting for age, gender, ADL and medical concerns). The Akaike information criterion (AIC) were obtained to compare non-nested logistic models. In order to evaluate the discriminant capacity of each model, the area under the curve (AUC) obtained from receiver operator characteristic (ROC) curves was used. Comparisons of ROC curves for the same adverse outcome were performed considering a proposed approach (Hanley and McNeil, 1983). The significance level was set at 0.05 for all analysis.

## Results

The sample comprises 4499 patients with a mean age of 76.3, standard deviation (SD) of 7.3 years, range 65–103 years. Of these 1854 (41.2%) were male and 2645 (58.8%) female. Only 734 patients (16.7%) were living alone. In total, 1616 (35.9%) of the patients were scored as having mental health concerns using the RISC, 2043 (45.4%) with ADL concerns and 3222 (78.3%) with medical concerns. Of patients registering mental health concerns on the RISC, less than half (48.5%) had a caregiver network that was perceived to be able to manage the situation. Option five of the care network (absence/liability) was not considered in the analysis because of the absence of records (**Table 1**). Evaluating the risk of adverse outcomes showed that 16.3% of the sample were perceived to be at risk of institutionalization, 32.8% at risk of hospitalization and 23.1% at risk of death.

**Table 1:** Characteristics of the sample.

	*n*	%
**Gender**
Male	1854	41.2
Female	2645	58.8
Age in years [mean(SD)]	76.3 (7.3)
**Living alone**
Yes	734	16.7
No	3653	83.3
**Mental health concerns**
No	2883	64.1
Yes	1616	35.9
**Severity**
Mild	847	52.4
Moderate	559	34.6
Severe	210	13.0
**Caregivers ability to manage mental health concerns**
Can manage	783	48.5
Carer strain	531	32.9
Some gaps	221	13.7
Cannot manage	81	5.0
Absent/liability	0	0.0
**ADLs concerns**
No	2456	54.6
Yes	2043	45.4
**Medical concerns**
No	977	21.7
Yes	3222	78.3


### Predictive Factors of Mental Health Concerns

In order to identify potential predictive factors of mental health concerns, univariable logistic regression models were performed. Results are shown in **Table 2**. Females had greater odds of mental health concerns [odds ratio (OR) = 1.26, 95% confidence interval (CI) 1.11–1.43] compared with males. Patients with either ADL concerns (OR = 10.1, 95% CI 8.76–11.7) or medical concerns (OR = 6.01, 95% CI 4.88–7.44) also had greater odds of having mental health concerns. Increasing age increased the risk of having mental health concerns (OR = 1.08, 95% CI 1.07–1.09) while living arrangement was not related to mental health concerns.

**Table 2:** Univariable and multivariable logistic regression models of mental health concerns.

	*n*	%	Unadjusted			Adjusted		
				
			OR	95% CI	*p*	OR	95% CI	*p*
**Gender**								
Male	608	32.8	1	–	0.001	1	–	0.293
Female	1008	38.1	1.26	1.11–1.43		1.08	0.93–1.25	
Age, years	–	–	1.08	1.07–1.09	<0.001	1.03	1.02–1.04	<0.001
**Living alone**								
No	1313	35.9	1	–				
Yes	272	37.1	0.95	0.81–1.12	0.566			
**ADL concerns**							
No	344	14.0	1	–	<0.001	1	–	<0.001
Yes	1272	62.3	10.1	8.76–11.7		7.15	6.08–8.41	
**Medical concerns**							
No	108	11.1	1	–	<0.001	1	–	<0.001
Yes	1508	42.8	6.02	4.88–7.44		1.96	1.55–2.49	


Considering significant factors of mental health concerns obtained in univariable models, a multivariable logistic regression model was performed and all factors remained statistically significant with the exception of gender. This reinforces that the presence of ADL and medical concerns and older age were associated with higher odds of mental health concerns (**Table 2**).

### Mental Health as a Predictive Factor of Adverse Healthcare Outcomes

Three multivariable logistic regression models were performed for the risk of each adverse outcome (institutionalization, hospitalization, and death). Model 1 (M1) included the presence/absence of mental health concerns, model 2 (M2) included the severity of concerns and model 3 (M3) the ability of the caregiver network to manage risk. Gender, age, ADLs, and medical concerns were included as covariates. Results are presented in **Table 3**.

**Table 3:** Multivariable logistic regression models of risk of adverse healthcare outcomes.

Model	Mental health	Risk of institutionalization			Risk of hospitalization			Risk of death		
				
		OR	95% CI	AIC	OR	95% CI	AIC	OR	95% CI	AIC
M1	Concerns									
	No	1	–	3197.7	1	–	4319.5	1	–	3761.3
	Yes	2.22	1.83–2.69		1.66	1.41–1.94		1.69	1.42–2.01	
M2	Severity									
	No	1	–	3127.5	1	–	4260.9	1	–	3673.6
	Mild	1.42	1.13–1.80		1.17	0.97–1.41		1.11	0.90–1.36	
	Moderate	2.81	2.21–3.57		2.28	1.83–2.84		2.09	1.66–2.62	
	Severe	5.30	3.82–7.36		3.85	2.66–5.57		5.41	3.79–7.72	
M3	Caregiver									
	No	1	–	2984.8	1	–	4262.8	1	–	3697.1
	Can manage	1.07	0.84–1.38		1.26	1.04–1.52		1.25	1.01–1.55	
	Carer strain	2.11	1.65–2.71		1.65	1.32–2.06		1.80	1.43–2.28	
	Some gaps	8.25	5.93–11.5		3.35	2.39–4.69		2.15	1.57–2.95	
	Cannot manage	16.9	9.48–30.2		6.44	3.41–12.2		10.3	5.66–18.7	


*^∗^Models adjusted for gender, age, ADL, and medical concerns.*

All three predictive factors (living alone, ADL concerns, and Medical concerns) of the three adverse healthcare outcomes were statistically significant. Patients with mental health concerns had greater odds of perceived risk of each adverse outcome (OR = 2.22, 95% CI 1.83–2.69 for risk of institutionalization; OR = 1.66, 95% CI 1.41–1.94 for risk of hospitalization; OR = 1.69, 95% CI 1.42–2.01 for risk of death). Additionally, as the severity of concern increased, the odds of perceived risk also increased (OR for each level of severity – mild, moderate and severe – varies between 1.11 and 5.41), compared to those without mental health concerns. Finally, the odds of perceived risk of each adverse outcome increased as the ability of the caregiver network to manage mental health concern decreased (from ‘can manage,’ to ‘carer strain,’ ‘some gaps,’ to ‘cannot manage’): OR for each level of caregiver network varies between 1.07 and 16.9. Model 3 revealed a lower AIC for the risk of institutionalization, while model 2 showed a lower AIC for risk of hospitalization and death.

The results of ROC curve analysis are presented in **Table 4**. All models present an AUC greater than 0.80 (values varies between 0.810 and 0.838), suggesting good discriminatory capacity. Comparing the models for each outcome, only M1 was statistically different from the M3 considering perceived risk of institutionalization as outcome (AUC = 0.810 for M1 and AUC = 0.838 for M3, *p* = 0.005).

**Table 4:** Discriminatory capacity of all three model to predict risk of adverse healthcare outcomes (AUC and standard error).

Model	Risk of institutionalization		Risk of hospitalization		Risk of death	
			
	AUC (SE)	*p*^∗^	AUC (SE)	*p*^∗^	AUC (SE)	*p*^∗^
M1	0.810 (0.007)	0.116	0.816 (0.006)	0.234	0.819 (0.007)	0.080
M2	0.824 (0.007)	0.005’	0.823 (0.006)	0.405’	0.828 (0.007)	0.050’
M3	0.838 (0.007)	0.084”	0.821 (0.006)	0.999”	0.826 (0.007)	0.697”


*^∗^*p*: comparing M1 and M2; *p*’: comparing M1 and M3; *p*”: comparing M2 and M3.*

## Discussion

This study presents the prevalence of cases of mental health concerns (composite neurocognitive and mood disorders) in the selected study population, their severity, and the perceived ability of caregiver networks to manage and the perceived risk of adverse healthcare outcomes in a large sample of community-dwelling older adults in Portugal, scored by community healthcare professional using a new short, global risk assessment instrument, akin to a brief targeted geriatric assessment, called the RISC. The results show that 35.9% of older patients presenting to PC in northern Portugal are judged by their healthcare professional to have mental health concerns. The severity of these mental health concerns was generally mild (52.4%) although 34.6% were moderate and 13% severe. It is possible that those with mild mental health concerns do not fulfill the criteria to be diagnosed with mental health disorders but instead represent patients with subclinical mental health problems that nevertheless contribute to morbidity and mortality particularly in older people (Steffens et al., 2000; Xavier et al., 2013). The percentage of people aged 65 or more years with mental health problems, identified by the GPs in this study, is consistent with existing figures; the lifelong prevalence for mental health diseases in PC patients is estimated to be 42.7%, and the one year prevalence for the general Portuguese population is 22.6% (DGS, 2013).

Mental health concerns were higher in women, older people and people with physical and functional problems as expected but when the model was adjusted, gender was no longer significant suggesting that older age, disease and functional impairment are more relevant than gender itself.

In this study patients with mental health problems had high levels of comorbidity with 78% scored for physical and medical concerns using the RISC. ADL were also associated with mental health problems with 45.4% of the patients having functional impairment. This was found to increase perceived risk, as judged by healthcare professionals, of all three adverse healthcare outcomes of interest in this study: institutionalization, hospitalization, and death.

We found that the best predictors of mental health problems were older age, impairment in ADL and medical problems, stressing the relevance of functionality and also comorbidity in old age corroborating other studies (Paul et al., 2006; Lee and Lee, 2011; Veerbeek et al., 2014). Loss of independence in performing ADL is a major concern for older people impacting upon self-perceived quality of life (Fernandez-Ballesteros et al., 2010). The relevance of caregiver networks to patients with mental health problems is established in the literature (Carretero et al., 2009) supporting other studies that suggest that competent caregiving can prevent risk of adverse outcomes (Giles et al., 2004). This study shows that almost half (48.5%) of caregiver networks were perceived by healthcare professionals in PC to be able to manage mental health concerns; however, a further 32.9% were deemed under strain and 5% unable to manage. The strain or burden placed on caregivers significantly increased the probability of perceived adverse outcomes, particularly for risk of institutionalization.

By comparing models we verified that the risk of institutionalization was increased when the caregiver network was deemed unable to cope. This again corroborates several studies stressing the importance of caregiver burden and its clear deleterious effects on older people including quality of life and increasing the cost of health and social care provision (Schulz and Martire, 2004).

This study has a number of limitations. The design was not able to examine point prevalence, but only the frequency or prevalence of cases in the selected study population. Thus, the sample is not probabilistic given that it was dependent on the willingness of the existing PC teams in the region to participate, introducing possible selection bias. This bias, however, does not affect the population selected, as most patients do not choose a GP but are assigned to one who is available. Further, this population-based study covers the whole northern region of Portugal increasing the external validity of the results. This cross-sectional study does not allow us to draw conclusions on a causal relationship between variables. Mental health, physical health and function are highly related such that mental disorders can be precursors of other chronic disease as well as the consequence of them (WHO and the Calouste Gulbenkian Foundation, 2014). The interaction between these conditions must be considered in screening and treatment strategies. Finally, this study presents the results of perceived rather than the actual risk of adverse outcomes, as scored by the RISC. Given the paucity of valid and reliable risk-prediction instruments available for use in the community (O’Caoimh et al., 2015a), the RISC seems a reasonable choice. Similar to the initial validation study in Ireland (O’Caoimh et al., 2012, 2015c) a prospective study is now underway in Portugal to determine whether the health care professionals predictions, as scored by the RISC, were able to accurately predict each of the three adverse outcomes of interest (institutionalization, hospitalization, and death) in Portugal. One-year outcomes are expected soon and further studies will investigate the predictive validity of the RISC against other risk-prediction instruments. The RISC is also being investigated in other populations and countries including Spain and Australia.

## Conclusion

Awareness of the importance of screening and assessing mental health concerns in PC is increasing. The RISC is a short, subjective, global risk-prediction instrument that can be used to train healthcare professionals (GPs and nurses) to screen and triage older people at risk of adverse outcomes including those with mental health problems. This instrument, unlike many others, includes an assessment of the patients’ caregiver networks in its risk assessment algorithm (O’Caoimh et al., 2015a). Scoring the RISC is quick, suggesting that it can easily be incorporated into routine clinical practice. This study reaffirms the high prevalence of mental health disorders in PC, especially those with mild or subclinical syndromes, and highlights important factors such as age, and the presence of physical comorbidities and functional impairment, and the outcomes that they are perceived to predict. Given the current demand for services and the prevailing shortage of resources, the need to triage patients will continue. Early identification of mental health concerns in older patients in PC should allow healthcare professionals initiate prompt, appropriate and directed treatment. Further research is now required to confirm these findings.

## Author Contributions

CP and LT developed the study aim and design. LT undertook the analyses. MA, SA, and MD conducted the fieldwork and contribute to the discussion. ROC and WM developed the measurement instrument and trained the researchers. CP coordinated writing of the paper. All authors contributed to the final version.

## Conflict of Interest Statement

The authors declare that the research was conducted in the absence of any commercial or financial relationships that could be construed as a potential conflict of interest.
